# Supplementary material for: Construction of Co,N-Coordinated Carbon Dots for Efficient Oxygen Reduction Reaction
Source: Molecules. 2022 Aug 7;27(15):5021. doi: 10.3390/molecules27155021 (PMC9370474; doi:10.3390/molecules27155021)
Supplement: Supplementary file 1 [file molecules-27-05021-s001.zip › molecules-1836382-supplementary.pdf]

Supporting Information

Construction of Co,N-Coordinated Carbon Dots on Carbon Black for Efficient Oxygen Reduction Reaction

Mengying Le †, Bingjie Hu †, Meiying Wu, Huazhang Guo and Liang Wang *

Institute of Nanochemistry and Nanobiology, School of Environmental and Chemical Engineering, Shanghai University, 99 Shangda Road, BaoShan District, Shanghai, 200444, China

*Corresponding authors: wangl@shu.edu.cn
†These authors contributed equally to this work.

The supporting information file includes 4 Pages and 5 Figures.

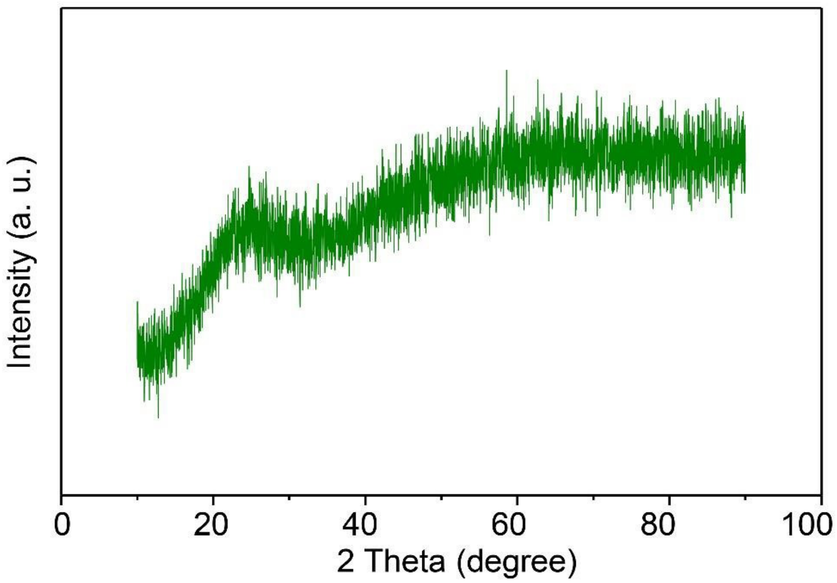

Figure S1 XRD pattern of Co-CDs.

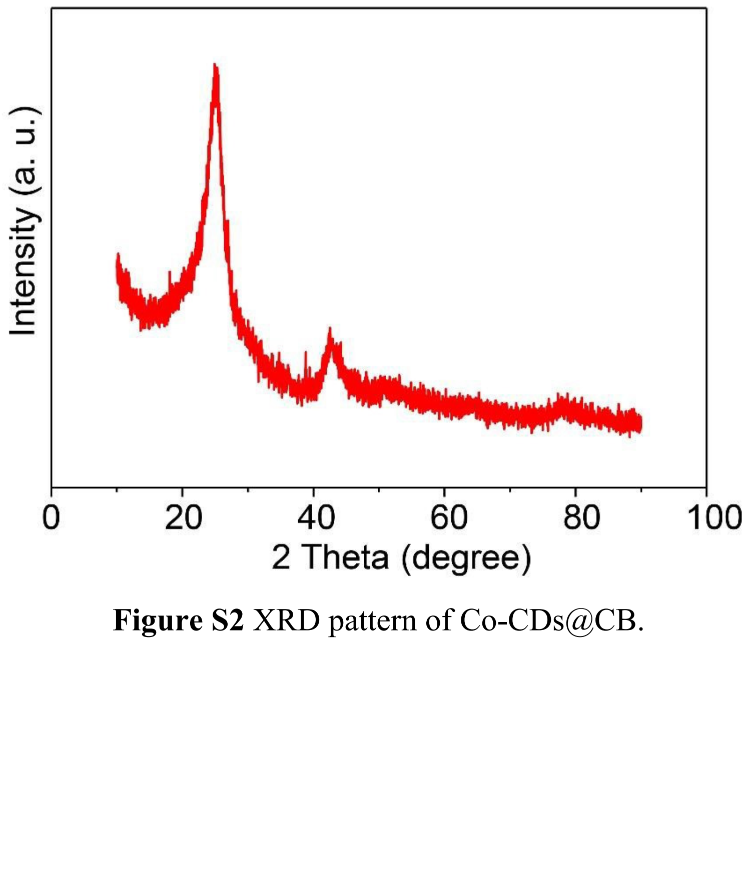

Figure S2 XRD pattern of Co-CDs@CB.

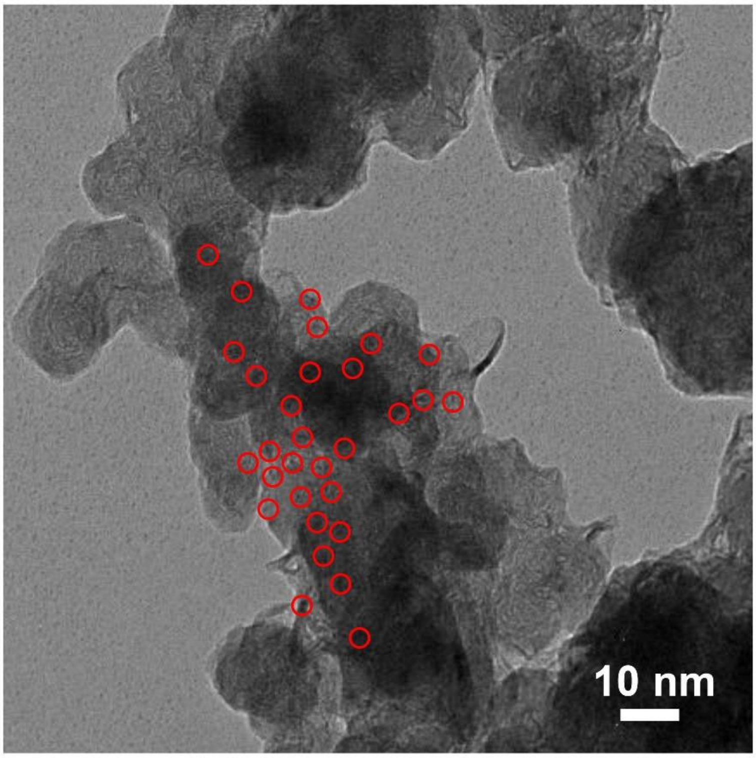

Figure S3 TEM image of Co-CDs@CB in x70.0 K version, the red circles represent the loaded Co-CDs.

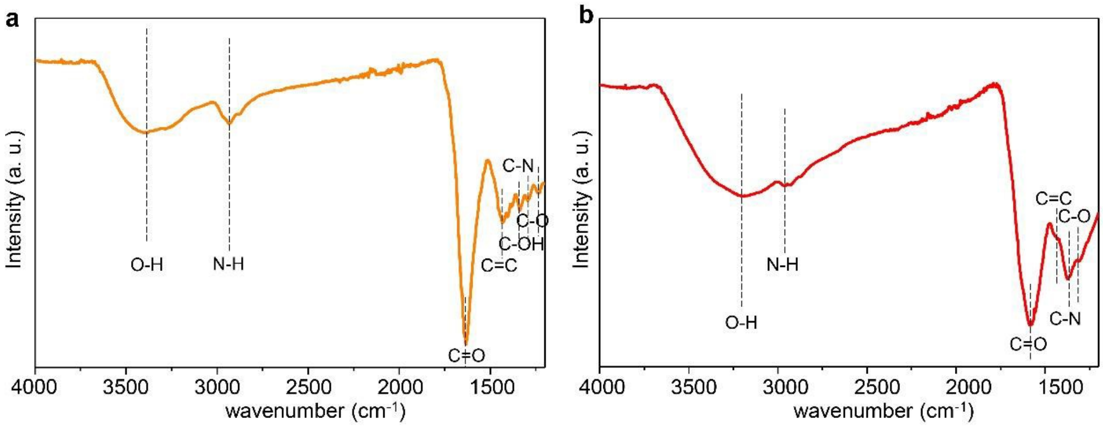

Figure S4 FTIR spectra of CDs and Co-CDs.

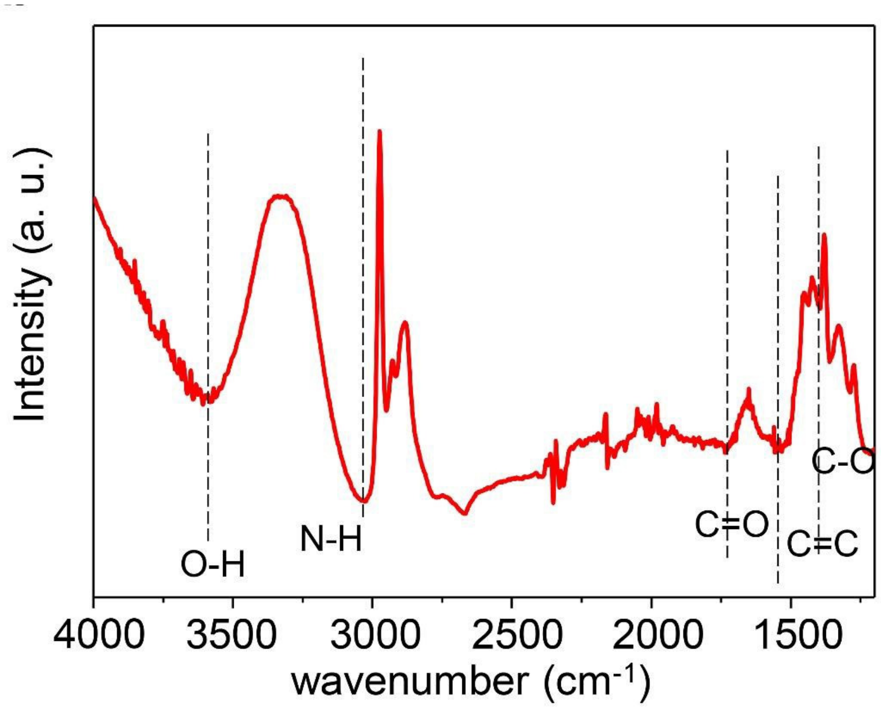

Figure S5 FTIR spectrum of Co-CDs@CB.
